# Meta-analysis of variance: an illustration comparing the effects of two dietary interventions on variability in weight

**DOI:** 10.1093/emph/eow020

**Published:** 2016-08-03

**Authors:** Alistair M. Senior, Alison K. Gosby, Jing Lu, Stephen J. Simpson, David Raubenheimer

**Affiliations:** ^1^Charles Perkins Centre; ^2^School of Mathematics and Statistics; ^3^School of Life and Environmental Sciences; ^4^Faculty of Veterinary Sciences, The University of Sydney, Sydney, New South Wales 2006, Australia

**Keywords:** effect size, diet, low carbohydrate, nutrition, obesity, standard deviation

## Abstract

Meta-analysis, which drives evidence-based practice, typically focuses on the average response of subjects to a treatment. For instance in nutritional research the difference in average weight of participants on different diets is typically used to draw conclusions about the relative efficacy of interventions. As a result of their focus on the mean, meta-analyses largely overlook the effects of treatments on inter-subject variability. Recent tools from the study of biological evolution, where inter-individual variability is one of the key ingredients for evolution by natural selection, now allow us to study inter-subject variability using established meta-analytic models. Here we use meta-analysis to study how low carbohydrate (LC) *ad libitum* diets and calorie restricted diets affect variance in mass. We find that LC *ad libitum* diets may have a more variable outcome than diets that prescribe a reduced calorie intake. Our results suggest that whilst LC diets are effective in a large proportion of the population, for a subset of individuals, calorie restricted diets may be more effective. There is evidence that LC *ad libitum* diets rely on appetite suppression to drive weight loss. Extending this hypothesis, we suggest that between-individual variability in protein appetite may drive the trends that we report. *A priori* identification of an individual’s target intake for protein may help define the most effective dietary intervention to prescribe for weight loss.

## Abstract (precis)

New meta-analysis methods from evolutionary biology allow us to ask how treatments affect variability, as opposed to just the average. Using these methods we demonstrate that low carbohydrate *ad libitum* diets may have more variable outcomes than calorie restricted diets.

## INTRODUCTION

When coupled with systematic review, meta-analysis is widely regarded as one of the most valuable tools available to guide evidence-based practices [1, 2]. Accordingly, almost every scientific field has now adopted meta-analytic approaches, and nutrition is no exception [e.g. 3]. In nutritional research meta-analysis is typically used to evaluate the efficacy of a dietary intervention by calculating an effect size that corresponds to the difference in the average weight (or weight loss) of groups of subjects on different diets within a study [e.g. 4, 5]. Although these meta-analyses provide important insights, by virtue of their focus on group averages they largely overlook between-subject variability in weight (although such variance does influence the standard error of the associated effect size). At best meta-analysis of dietary interventions make a statistical correction for differences in variance between groups (e.g. by using a standardized mean difference; [6]), but variance in weight itself is rarely, if ever treated as the primary outcome.

To-date there a have been a handful of instances in which meta-analyses have focused on variance as a primary outcome outside of nutritional research (e.g. [7–9]). However, in general the widespread adoption of meta-analysis of variability has been hampered by the lack of a formal framework that is well integrated with standard meta-analytic models. Such a framework was recently developed in evolutionary biology [10], a field where trait variation is considered as important as, or for some purposes even more important than, the trait mean. Darwin acknowledged the importance of intra-population variation as a central tenet of evolution by natural selection, and driver of adaptation, at the publication of the concept in 1859. In public health and nutrition, however, inter-subject variability has been largely treated as statistical noise, with little regard for its biological significance.

From an applied perspective understanding how any intervention affects variability in an outcome is just as important as understanding its effects on the mean, and our focus on the latter hinders our ability to truly understand treatment efficacy [11]. For instance, if a diet ubiquitously reduces the average weight of a group, but in fact consistently causes weight gain in a fraction of the subjects, then the mean response cannot be regarded as representative of all individuals and it is erroneous to conclude that the diet is an effective treatment for the whole population. Yet, it may be tempting to infer such a conclusion from a mean-focused meta-analysis of such data. What is more, given modern-medicine’s pursuit of personalized health-care [12], and an ever-increasing appreciation of the role of the interaction between an individual’s genetics and their environment in governing obesity risk [13], understanding which dietary interventions elicit a high degree of between-subject variability in the response is more important than ever.

The most simplistic methods proposed by Nakagawa *et al.* [10] require nothing more than a reformulation of the effect-size, and use exactly the same raw statistics as would normally be collected for meta-analysis of the mean (i.e. mean, standard deviation and sample size). Outside of public health these tools have now been applied to understand variability in phenomena as diverse as decision-making, the effects of sex-hormones on immune function, the evolution of dietary niche and even the biology of ageing [14–17].

Substantial effort has gone into testing how dietary macronutrient composition contributes to weight loss. Given that dietary macronutrient composition is vital for control of appetite and energy intake [18, 19], and that nutritional appetites may differ between individuals depending on cultural backgrounds and other life experiences (e.g. *in utero* and early-life environments; [20]), it is of interest to compare the variance in the mass of subjects on different dietary regimes. Two popular dietary regimes are *ad libitum* low carbohydrate (LC) and calorie restriction (CR) diets. LC diets with unrestricted protein and fat intakes rely upon macronutrient composition, possibly ketosis and reduced food variety to increase satiety and reduce energy intake for weight loss [21, 22]. In contrast, CR protocols rely on the individual following a prescribed level of calorie-restriction, rather than responding *ad libitum* to appetite signals. Given that LC diets rely to a greater extent than CR diets on the physiological signals of individual subjects to drive energy intake, we predict that these diets may generate more variability in weight than diets that prescribe a CR. Here, we use meta-analytic models to compare variability in body mass after 6 months of LC *ad libitum* interventions to 6 months of CR using studies identified in a recent to-date systematic review [4].

## METHODS

### Data collection

We started with the library of studies from which Tobias *et al.* [4] extracted the data in their analyses. To be included in our analyses the study was required to contain at least one diet that restricted calorie intake, and one diet that restricted carbohydrate intake but allowed subjects to eat *ad libitum*; 7 studies met our criteria (Table 1). Most studies contained one group of subjects on each type of diet, although Gardner *et al.* [23] and Shai *et al.* [24] contained two groups on different types of calorie-restriction. This gave us data from 16 groups of subjects on dietary interventions; 7 LC diets and 9 on CR diets. From each group we extracted the mean and SD in mass (kg) of individuals, and sample sizes, on each diet after 6 months.

**Table 1.** Details of the studies included in our analysis, and the baseline characteristics of subjects in the LC *ad libitum* and CR groups

ID	Reference	Baseline mass (Kg) mean ± SD (*n*)	Population characteristics	Country	Calorie-restricted diet (additional to CR)	*Ad libitum*, LC-restricted
1	Dansinger *et al.* [46]	LC: 100 ± 14 (40)	At risk for cardiovascular disease	USA	Weight-watchers	Atkins
		CR: 97 ± 14 (40)				
2	Foster *et al.* [47]	LC: 100 ± 20 (33)	Obese	USA	Low-fat (25%)	Atkins
		CR: 99 ± 16 (30)				
3	Foster *et al.* [48]	LC: 103 ± 16 (153)	Obese	USA	Low-fat (30%)	Atkins
		CR: 106 ± 14 (154)				
4	Gardner *et al.* [23]	LC: 86 ± 13 (77)	Overweight pre-menopausal women	USA	LEARN; Zone (30% fat)	Atkins
		CR: 85 ± 14 (79)				
		CR: 86 ± 10 (76)				
5	Iqbal *et al.* [49]	LC: 118 ± 21 (70)	Obese with type-2 diabetes	USA	Low-fat (30%)	Low-carbohydrate
		CR: 116 ± 17 (74)				
6	Samaha *et al.* [50]	LC: 130 ± 23 (64)	Obese	USA	NHLBI (30% fat)	Low-carbohydrate
		CR: 132 ± 27 (68)				
7	Shai *et al.* [24]	LC: 92 ± 14 (109)	Type-2 diabetes, cardiovascular disease or obese	Israel	American Heart Association (30% fat); Mediterranean Diet (30% fat)	Atkins
		CR: 91 ± 12 (104)				
		CR: 91 ± 14 (109)				

Our library of studies was collected from the library of studies outlined in Table 1 of Tobias *et al.* [4].

Where results were reported graphically we extracted data using GraphClick [25]. In three studies results were reported as the mean and SD in mass at baseline and the change in mass from baseline at 6 months. In such cases we calculated the mean mass at 6 months by adjusting the mean at baseline by the mean change. The SD in mass at 6 months was calculated as the square root of the sum of the variance in mass at baseline and that in the change in mass (i.e. propagation of error). This protocol assumes that there is no correlation between initial mass and change in weight at 6 months. Where they are known to exist such correlations can be accounted for, however no such correlation was mentioned in those studies in our dataset to which this protocol was applied. Furthermore, there is no widespread evidence for such an association in the literature [26]. Where possible we used population-level statistics that excluded dropouts. In one case we had to use data where baseline characteristics were carried-forward for dropouts [23], although in all instances effects size weighting was based on the number of actual participants in the trial at 6 months (the more conservative approach).

### Effect-sizes and statistical models

**Figure 1.**
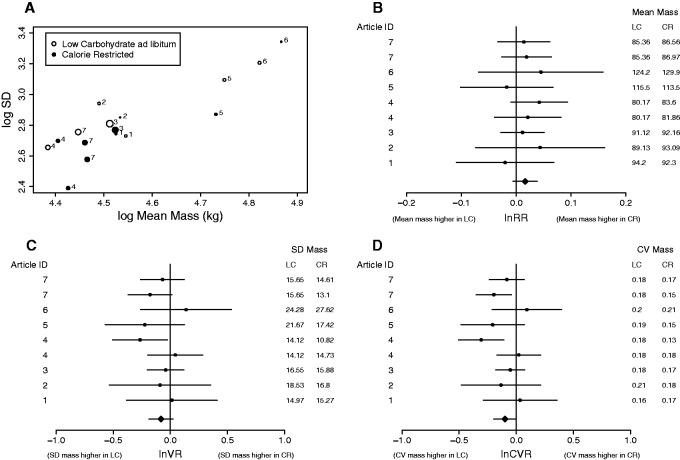
**(A)** The log mean and log SD mass (kg) after 6 months on a high protein, carbohydrate-restricted *ad libitum* (open) or calorie-restricted (solid) diet as reported in those published studies included in our analyses. The size of the point corresponds to the precision (1/sampling error for lnSD) of the effect size. Forest plots for **(B)** lnRR, **(C)** lnVR and **(D)** lnCVR. Round points give effects sizes calculated from each study, and bars the associated 95% confidence limits. Mean effects as estimated by multi-level meta-analysis are shown as diamonds at the bottom of the plot. To right of each panel is the relevant statistic for the two groups included in the effect size. In all panels numbers correspond to article IDs as given in Table 1

Differences in the mean mass of groups on different diets within studies were quantified as the log of the ratio of the mean in each group, also known as the log response ratio (lnRR and its sampling variance, $s_{\text{lnRR}}^{2}$; Table 2). To analyze differences in variance, Nakagawa *et al.* [10] suggest several methods, which differ in the way that concurrent changes in the mean and variance are accounted for. Many biological systems seem to follow a mean–variance relationship sometimes termed Taylor’s Law; an empirically derived relationship which states that as the mean increases, the variance also increases following a power relationship [10]. Given this expected relationship, it may be most meaningful to ask whether the variance of two groups differs, after accounting for differences in the mean. Collectively our data appear to show a linear relationship between log SD and log mean mass, as would be expected based on Taylor’s Law (Fig. 1A). However, within-studies a positive relationship between log SD and log mean mass was not consistently observed; e.g. in Sahi *et al.* [24] there is an apparent negative relationship (Fig. 1A).

**Table 2.** The effect sizes used, and their sampling variance, based on Nakagawa *et al.* [10]

Abbreviation	Primary Outcome	Effect Size	Sampling Variance (*s*^2^_Effect Size_)	Model
lnRR	Mean	$\text{ln}\left(\frac{{\bar{x}}_{E}}{{\bar{x}}_{C}}\right)$	$\frac{s_{C}^{2}}{n_{C}{\bar{x}}_{C}^{2}}+\frac{s_{E}^{2}}{n_{E}{\bar{x}}_{E}^{2}}$	Contrast-Based
lnVR	Standard Deviation	$\text{ln}\left(\frac{s_{E}}{s_{C}}\right)+\frac{1}{2(n_{E}-1)}-\frac{1}{2(n_{C}-1)}$	$\frac{1}{2(n_{C}-1)}+\frac{1}{2(n_{E}-1)}$	Contrast-Based
lnCVR	Coefficient of Variance	$\text{ln}\left(\frac{{\text{CV}}_{E}}{{\text{CV}}_{C}}\right)+\frac{1}{2(n_{E}-1)}-\frac{1}{2(n_{C}-1)}$	$\begin{array}{l} \frac{s_{C}^{2}}{n_{C}{\bar{x}}_{C}^{2}}+\frac{1}{2(n_{C}-1)}-2\rho \sqrt{\frac{s_{C}^{2}}{n_{C}{\bar{x}}_{C}^{2}}\frac{1}{2(n_{C}-1)}}\\ +\frac{s_{E}^{2}}{n_{E}{\bar{x}}_{E}^{2}}+\frac{1}{2(n_{E}-1)}-2\rho \sqrt{\frac{s_{E}^{2}}{n_{E}{\bar{x}}_{E}^{2}}\frac{1}{2(n_{E}-1)}} \end{array}$	Contrast-Based
ln$\bar{x}$	Mean	$\text{ln}\bar{x}$	$\frac{s^{2}}{n{\bar{x}}^{2}}$	Arm-Based
lnSD	Standard Deviation	$\text{ln}s+\frac{1}{2(n-1)}$	$\frac{1}{2(n-1)}$	Arm-Based

$\bar{x}$ is the group average mass, *s* is the group SD in mass, *n* is sample size, CV is the coefficient of variance, *ρ* is the correlation between ln$\bar{x}$ and lnSD. Where subscripts are included *C* and *E* were treated as the LC and CR groups, respectively, in our analyses. The type of model used to analyze each effect size is also given.

We explored three different methods for meta-analyzing variance. First, for each possible combination of diet types within a study we calculated the log variance ratio (lnVR) and its associated sampling variance ($s_{\text{lnVR}}^{2}$; Table 2), an effect size that assumes that there is no mean-variance relationship. Second we calculated the log of the coefficient of variance ratio (lnCVR) and its sampling variance ($s_{\text{lnCVR}}^{2}$; Table 2), which assumes there is a linear relationship between the mean and variance on the natural scale (note Taylor’s Law predicts a power relationship on the natural scale). Because both of these effect sizes and also lnRR (for mean mass) are effect sizes that correspond to relative differences between treatments within studies, they were analyzed using a conventional ‘contrast-based’ model [27, 28]. We used multi-level meta-analyses (MLMA), which included a random-factor accounting for the fact that some effect sizes arise from the same study, and a covariance matrix giving the expected covariance between those effect-sizes that are based on contrasts with the same LC dietary group; i.e. stochastic dependency [29] (see Supplementary Materials S1). All analyses were performed using the rma.mv function in the package ‘metafor’ in the statistical programming environment *R* version 3.2.1 [30, 31]. In all cases we consider estimates with a lower to upper 95% confidence limit (LCL to UCL) not spanning zero statistically significant. Data and code can be found on the online repository Dryad [32].

Finally, we explored an alternative approach where rather than calculate an effect size that corresponds to a contrast between groups within the same study, the outcome for each treatment group is analyzed directly, with differences between groups made using moderator variables; sometimes referred to as an ‘arm-based’ meta-analysis [27, 28]. In this method the log of the SD (adjusted for sample size, lnSD; Table 2), along with its sampling variance ($s_{\text{lnSD}}^{2}$; Table 2), was calculated for each group within a study. Following similar notation to that in Nakagawa *et al.* [10], differences between groups were then analyzed using multi-level meta-regression (MLMR) as described in equations (1)–(4):
(1)
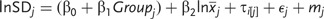

(2)
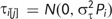

(3) $$\epsilon_{j}∼N(0,\sigma_{\epsilon }^{2}),$$

(4) $$m_{j}∼N(0,\sigma_{\text{ln}\sigma_{j}}^{2}),$$

where lnSD_*j*_ is the *j*th lnSD in the set of *n* effect sizes (*j* = 1, 2, … *n*), *Group_j_* is a dummy variable denoting whether the *j*th estimate comes from an LC (0) or a CR (1) diet, ln${\bar{x}}_{j}$ is the log of the mean mass of the *j*th group (transformed to a *Z*-score for model fitting), *β*_0_ is the overall intercept (here the average lnSD for LC diets), *β*_1_ is the coefficient for *Group* (here the average difference in lnSD between CR and LC diets), *β*_2_ is the coefficient for the effect of mean mass on lnSD, *τ*_[*i*]*j*_ is the random effect for the *j*th effect size in the *i*th study in the set of *k* studies (*i* = 1, 2, …, *k*), which is distributed following equation (2) (alternatively expressed as $\tau_{[i]j}=N(0,\sigma_{\tau }^{2}\text{diag}({\text{P}}_{1},{\text{P}}_{2},\ldots ,{\text{P}}_{k})$) where within the *i*th study, *τ*_[*i*]*j*_ is multivariate normally distributed with a mean of 0 and co-variance of $\sigma_{\tau }^{2}{\text{P}}_{i}$ (P_*i*_ is a correlation matrix with the off-diagonal elements being a common value of *ρ*, which is estimated by the model; i.e. effect sizes from different treatment groups within the same study are assumed to be correlated with one another with *ρ*), *ϵ_j_* is the residual for the *j*th lnSD, which is distributed following equation (3), and *m_j_* is the sampling error for the *j*th group, which is distributed following equation (4) (with the sampling variance fitted as $s_{\text{lnSD}}^{2}$ for the *j*th lnSD). The advantage of this approach over lnVR and lnCVR is that we are not forced to make rigid assumptions about the association between group mean mass and group variance in mass, as the strength of this relationship is estimated directly from the data (*β*_2_). In addition, because we do not calculate contrasts between dietary treatments there is no stochastic dependency (Supplementary Material S1). A potential drawback of this method is that the degree to which data from the same study are correlated with one another is also estimated from the data (*σ_τ_*^2^ and *ρ*), with the possibility that the study-level effect may be estimated as 0, leaving data points from the same study essentially independent. Where this occurs it may be considered a violation of the concurrent-control principle of meta-analysis [27, 33]. Further, the model described above is relatively complex and may suffer from over-parameterization when the number of effect sizes is limited, as is the case in the current analysis (which we explored using the profile function in ‘metafor’; [31]). Differences in the mean of two or more treatment groups may also be assessed using an arm-based model by fitting ln${\bar{x}}_{j}$ as the response along with its associated sampling variance ($s_{\text{ln}\bar{x}}^{2}$; following [10]). In this case the model could be implemented without estimation of *β*_2_, which would make the coefficient for Group (*β*_1_) similar to lnRR [34, 35]. Alternatively, lnSD may be fitted as the predictor where one wishes to determine and potentially correct for a mean-variance relationship; this becomes similar to using a standardized mean difference such as Hedge’s *d*, although it should be noted this is on a log scale [6].

## RESULTS

MLMA of lnRR estimated a small positive mean effect (amounting to a 2% difference between the mean of the two groups), and this difference was not-statistically significant (MLMA lnRR = 0.02, LCL to UCL = −0.01 to 0.04, Figure 1B; for full output from all models see Supplementary Material S2). Meta-analysis of lnVR estimated a negative mean effect, which amounted to the SD in mass being 8% lower on CR than LC diets, although the confidence limit for this estimate spanned zero indicating a non-significant difference (MLMA lnVR = -0.08, LCL to UCL = −0.19 to 0.02, Fig. 1C). Finally, MLMA of lnCVR detected a negative effect suggesting the coefficient of variance was lower for LC than CR groups. Again this effect was non-significant, although the UCL for this estimate was close to zero (MLMA lnCVR = −0.10, LCL to UCL = −0.20 to 0.90 × 10^−3^, Fig. 1D); the exponent of this estimated mean effect suggests that the coefficient of variance in the CR group is around 10% lower than that in the LC group. The average mean-corrected lnSD of groups after 6 months on a CR diet as estimated by MLMR (equation 1) was 2.77, whereas that on a LC *ad libitum* diet was 2.88, and the difference between the two was statistically significant (MLMR *β*_1_ = -0.11, LCL to UCL = -0.21 to -0.02). This result corresponds to the mean-corrected SD (the exponent of lnSD) of mass on CR diets being on average 10.5% lower than that on the LC diet, an overall effect magnitude similar to that estimated by MLMA of lnVR and lnCVR. MLMR estimated a significant positive slope of log mean mass on lnSD (MLMR *β*_2_= 0.20, LCL to UCL = 0.14–0.26).

**Figure 2.**
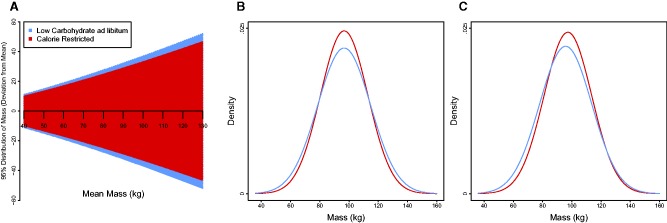
**(A)** Shaded areas give the predicted 2.5–97.5 percentiles in mass (kg) for a given mean mass (kg), as predicted by the MLMR of lnSD assuming a normal distribution. **(B)** Predicted probability densities for mass after 6 months on each diet based on the SD estimated by MLMR of lnSD, assuming a mean mass of 96.7 kg (the mean mass of all subjects as estimated by meta-analysis). **(C)** Predicted probability densities for mass after 6 months on each diet based on the SD estimated by MLMR of lnSD, assuming a different mean mass on each diet. For full MLMR coefficients see Supplementary Material S2

Using our MLMR estimates, and making the assumption that mass is normally distributed, it is possible to predict the entire distribution of weights for a group of subjects on each diet type; note that with a mean focused meta-analysis one would be restricted to predicting the mean of each group, alone. For a given mean mass, mass is predicted to be more variable in those groups on LC diets, than those on CR diets (Fig. 2A). For instance, if we assume a group with a mean mass of 96.7 kg (the average mass of all groups in our dataset as estimated by meta-analysis), a group on a LC diet is more likely to contain both individuals with mass >120 kg, and mass <80 kg than a group on a CR diet; see Figure 2B for the predicted probability density function of each group. These findings suggest that whilst LC diets are more effective than the alternative at generating lower weights in some individuals, this is not the case for the population as a whole.

It has been argued that LC *ad libitum* diets reduce mean mass more effectively than CR diets [5], making comparisons of distributions with an equal mean mass unrealistic; note that we have limited evidence for this in our own dataset (e.g. Fig. 1B). By combining the estimates from MLMR of the difference between ln$\bar{x}$ in LC and CR groups (see Supplementary Material S2) and MLMR of lnSD, we can generate estimated probability density functions for each diet that account for differences in mean and SD in mass simultaneously (Fig. 2C). Assuming that the estimated difference in mean mass between the two diets is accurate, the predicted distributions yield two insights. First, groups on LC *ad libitum* diets have a slightly higher probability (0.62%) of containing subjects with mass >125 kg than CR diets (Fig. 2C). Although this probability seems small, over an entire population there could be a substantial number of overweight people (>1 in every 200) who would have a lower weight after 6 months on a CR diet than an LC diet. Secondly, groups on LC diets have a substantially higher probability of containing subjects with mass <80 kg than CR diets (Fig. 1D). This latter artifact, however, arises by virtue of the fact that LC diets simultaneously have a higher SD and lower mean mass (albeit by a modest amount) than the alternative, and would be overlooked if we solely focused on differences in mean mass.

## DISCUSSION

Using a recently developed framework for meta-analysis of variability we present evidence for a greater variation in body mass following a LC *ad libitum* intervention in comparison to a CR protocol, despite a slight (non-significant) trend for lower mean body mass following the former. Although the sign and magnitude of the difference in variability in mass between groups was relatively consistent, the effect was not significant in all analyses. In particular, the precision of the associated difference between groups was influenced by the way in which correction for a mean-variance relationship was made. Analyses of lnVR, which is independent of between-treatment differences in mean mass had a very wide confidence limit. However, lnCVR and arm-based models, which made correction for differences in group means, identified more precise effects. Taken collectively, our data certainly suggest a mean-variance relationship; however, at the within-study level this relationship was not consistently observed (a potential example of Simpson’s paradox; [36]). Nevertheless, our work illustrates the importance of simultaneously analyzing variance- and mean-focused effect sizes in nutritional meta-analyses. No study has performed a full analysis of means and variance associated with all dietary and lifestyle interventions used for weight loss, but here we have provided preliminary insights into differences in effectiveness between LC and CR protocols using the meta-analysis of variance method described by Nakagawa *et al.* [10].

The LC interventions in our dataset used the Atkins protocol or similar, which prescribes a reduced carbohydrate intake without a restriction on protein or fat intakes leading to a 6 month diet that is relatively high in percent protein and fat (22% protein, 47% fat and 28% carbohydrate; [23]). Protein-induced satiety is a key driver of reduced energy intake, which in turn promotes weight loss on a LC diet. Variance in this response may explain the greater variance in body mass at 6 months on the LC diet in comparison to the set energy intake of CR interventions.

**Figure 3.**
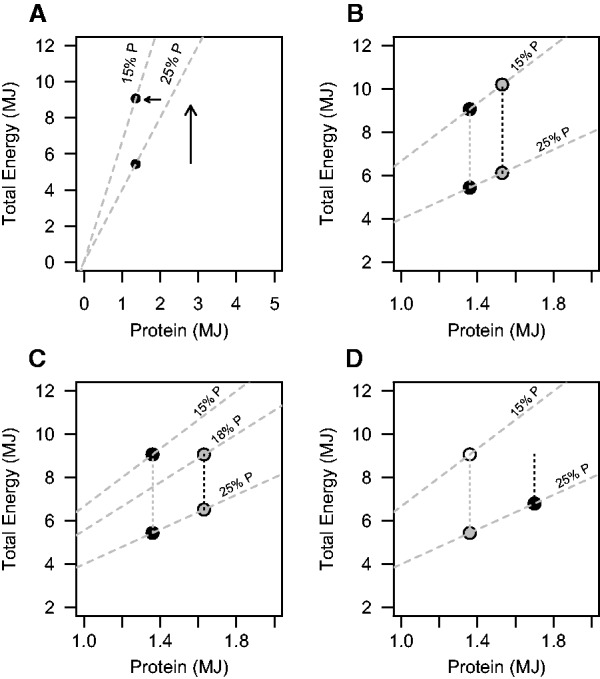
Protein and total energy (mega joules; MJ) intake plotted along the *x*- and y-axes, respectively. Grey dashed lines represent nutrient rails reflecting diets with differing average percent protein (%P). Points denote the amount of energy an individual consumes on a diet (i.e. their appetite). **(A)** Protein leverage dictates that as the proportion of dietary protein decreases (e.g. from from 25%P to the 15%P; small arrow), total energy intake increases (black points and large arrow) to maintain absolute protein intake relatively constant. **(B)** There may be between-individual variance in absolute protein targets. Accordingly, for a given %P, individuals with a higher protein target (grey circle) consume more total energy than those with a lower protein target (black circle). **(C)** An individual with a high protein target but a lower %P in their habitual diet (grey points) may experience a smaller reduction in energy intake (black dotted line) when %P increases, than an individual with a lower protein target and %P in their habitual diet (black point, grey dotted line). **(D)** There may be variation in the strength of protein leverage. Thus, on a diet with a low %P, individuals may have similar intakes (white point). However, as %P increases some individuals may maintain constant protein intake (grey point), where as others may over-consume protein (black point; e.g. to satisfy an appetite for carbohydrates). Figures redrawn from Simpson and Raubenheimer, which the reader should see for a more detailed examination of protein leverage see [37]

Simpson and Raubenheimer proposed a key role for protein appetite in driving the human obesity epidemic—the protein leverage hypothesis (Fig. 3A; [37]). Population studies, large dietary trials, experimental studies and synthesis of 38 published experimental trials show that humans prioritize, or ‘defend’, protein intake at the expense of regulation of carbohydrate and fat intake [18, 38–41]. Therefore, when the percentage of protein in the diet is reduced total energy intakes increase to maintain absolute protein intake relatively constant (Fig. 3A). Protein appetites may differ between individuals through differences in the absolute protein target (Fig. 3B and C) and the strength of protein leverage (i.e. variance in the range of absolute protein intakes an individual consumes when percent dietary protein changes; Fig. 3D). Protein appetites may vary due to different cultural backgrounds, *in utero* experiences or disease states such as insulin resistance [20, 37].

As well as these environmental factors, it seems reasonable to assume that there is a genetic contribution in the degree to which specific macronutrients are regulated. Cross taxa comparisons demonstrate that the degree to which the intake of specific macronutrients is regulated differs between closely related species implying that such traits are evolutionarily labile and populations may contain heritable variation. In the case of primates, on average humans appear to regulate protein intake at the expense of overconsumption of non-protein energy, yet Gorillas (*Gorilla gorilla*) regulate carbohydrate energy intake [19]. Regulatory priorities of other nutrients such as carbohydrate may also contribute to determining the success of diets differing in nutrient composition. For instance, if humans have a specific appetite for carbohydrate, similar to numerous other species, including mice [41], the suppressive effect of protein on appetite and energy intake may be dampened by compensatory intake of carbohydrate on LC diets.

An individual’s protein target and strength of protein leverage could be used to predict success on weight loss regimes that rely upon feedback from protein appetite to drive reduced energy intake [20, 37]. Figure 3B–D presents scenarios that describe how differences in protein targets and the strength of protein leverage may impact the success of a LC diet. For instance, the magnitude of an individual’s absolute protein target may interact with the percentage protein in their habitual diet to govern the net reduction in calories consumed when percentage dietary protein increases on an LC *ad libitum* diet (Fig. 3B and C). Determining predictors of success on LC diets might not only be important from a weight management perspective. Studies on model organisms and data from human trials and cohort studies suggest that diets high in protein and low in carbohydrate content are associated with decreased longevity and poor late-life health outcomes [43, 44]. Thus these interventions should perhaps only be prescribed when they are likely to have substantial beneficial impact on weight.

The results of this study are limited to a 6-month period and adherence to a prescribed intervention may deteriorate beyond this point [23], attenuating long-term maintenance of weight loss in response to any dietary regime [45]. Irrespective of dietary prescription, the success of weight loss will depend upon appetite physiology, motivation and life experiences, and these factors are likely to vary between individuals. This variability should be explored. As above, we reiterate that the methods we use here require essentially the same summary statistics as are required for meta-analysis of the group mean [10]. Therefore, much of the data required for completion of a meta-analysis of variance to understand differences in success of various weight loss protocols may be available in published literature and could provide substantial advances in personalized weight management regimes. In cases where data sets are sufficiently large enough (note that our sample sizes here are relatively limited) moderator variables can be used to explore how the specifics of each study contribute to the magnitude of the observed variance as one would with meta-analysis of the mean. In the case of our analyses, we detected some heterogeneity in the meta-analysis of lnCVR (Supplementary Material S2). The macronutrient content of the LC and CR diets in our dataset differed slightly between studies, as did exercise recommendations (see Table 1). Thus, as more data become available one may use meta-regression to ask how differences in the macronutrient profile of diets and or prescribed exercise regimes contribute to differences in variability in mass.

Here, we provide an example to illustrate the importance of meta-analysis of variance in interpreting outcomes of dietary prescriptions used for weight management. In particular, it seems that individual variance in appetite response to dietary macronutrient composition may be vital in identifying the potential success an individual may experience on an intervention. The implications for assessing variability across different dietary interventions are substantial and could result in targeting phenotypes with specific weight loss interventions, improve our understanding of factors involved in appetite and body weight maintenance, and inform future study design.

## Supplementary Material

Supplementary Data

## References

[1] Liberati A Altman DG Tetzlaff J The PRISMA statement for reporting systematic reviews and meta-analyses of studies that evaluate health care interventions: explanation and elaboration. PLoS Med 2009; 6:e1000100. 19621070 10.1371/journal.pmed.1000100 PMC2707010

[2] Higgins JPT Green S. Systematic reviews and the Cochrane Handbook In: Higgins JPT Green SS (eds). Cochrane Handbook for Systematic Reviews of Interventions. Chichester, UK: John Wiley & Sons, Ltd, 2006.

[3] Axelson ML Federline TL Brinberg D. A meta-analysis of food- and nutrition-related research. J Nut Ed 1985;17: 51–4.

[4] Tobias DK Chen M Manson JE Effect of low-fat diet interventions versus other diet interventions on long-term weight change in adults: a systematic review and meta-analysis. Lancet Diabetes Endocrinol 2015;3:968–79. 26527511 10.1016/S2213-8587(15)00367-8 PMC4667723

[5] Nordmann AJ Nordmann A Briel M Effects of low-carbohydrate vs low-fat diets on weight loss and cardiovascular risk factors. Arch Intern Med 2006;166:285–932 16476868 10.1001/archinte.166.3.285

[6] Nakagawa S Cuthill IC. Effect size, confidence interval and statistical significance: a practical guide for biologists. Biol Rev 2007;82:591–605. 17944619 10.1111/j.1469-185X.2007.00027.x

[7] Raudenbush SW Bryk AS. Examining correlates of diversity. J Educ Stat 1987;12:241–69.

[8] Bond CF Jr DePaulo BM. Individual differences in judging deception: accuracy and bias. Psychol Bull 2008;134: 477–92. 18605814 10.1037/0033-2909.134.4.477

[9] Pigott TD Wu M-J. Methodological issues in meta-analyzing standard deviations: Comment on Bond and DePaulo. *Psychol Bull* 2008;134: 501–3. 10.1037/0033-2909.134.4.498 18605816

[10] Nakagawa S Poulin R Mengersen K Meta-analysis of variation: ecological and evolutionary applications and beyond. Methods Ecol Evol 2015;6:143–52.

[11] Christakis NA. The Average In: Brockman JS (ed)*.* This Idea Must Die: Scientific Theories that are Blocking Progress. New York: Harper Perennial, 2015, 532–4.

[12] Miles A Loughlin M Polychronis A. Evidence-based healthcare, clinical knowledge and the rise of personalised medicine. J Eval Clin Pract 2008;14:621–49. 19018885 10.1111/j.1365-2753.2008.01094.x

[13] Walley AJ Blakemore AIF Froguel P. Genetics of obesity and the prediction of risk for health. Hum Mol Genet 2006;15(suppl 2):R124–30. 16987875 10.1093/hmg/ddl215

[14] Simons MJ. Questioning causal involvement of telomeres in aging. Ageing Res Rev 2015;24:191–6. 26304838 10.1016/j.arr.2015.08.002

[15] Foo YZ Nakagawa S Rhodes G The effects of sex hormones on immune function: a meta-analysis. Biol Rev 2016; doi: 10.1111/brv.12243. 10.1111/brv.12243 26800512

[16] Charpentier E Looten V Fahlgren B Meta-analytic estimation of measurement variability and assessment of its impact on decision-making: the case of perioperative haemoglobin concentration monitoring. BMC Med Res Methodol 2016;16:1. 26787309 10.1186/s12874-016-0107-5 PMC4717612

[17] Senior AM Nakagawa S Lihoreau M An overlooked consequence of dietary mixing: a varied diet reduces inter-individual variance in fitness. Am Nat 2015;186:649–59. 26655777 10.1086/683182

[18] Gosby AK Conigrave AD Raubenheimer D Protein leverage and energy intake. Obes Rev 2014;15:183–91. 24588967 10.1111/obr.12131

[19] Simpson SJ Raubenheimer D. The Nature of Nutrition: A Unifying Framework from Animal Adaptations to Human Obesity. Oxford, UK: Princeton University Press, 2012.

[20] Raubenheimer D Machovsky-Capuska GE Gosby AK The nutritional ecology of obesity: from humans to companion animals. Br J Nutr 2015;113:S26–39. 25415804 10.1017/S0007114514002323

[21] Westerterp-Plantenga MS LG G Westerterp KR. Dietary protein – its role in satiety, energetics, weight loss and health. Br J Nutr 2012;108:S105–12. 23107521 10.1017/S0007114512002589

[22] Gibson AA Seimon RV Lee CMY Do ketogenic diets really suppress appetite? A systematic review and meta-analysis. Obes Rev 2015;16:64–76. 25402637 10.1111/obr.12230

[23] Gardner CD Kiazand A Alhassan S Comparison of the Atkins, Zone, Ornish, and LEARN diets for change in weight and related risk factors among overweight premenopausal women: the A TO Z Weight Loss Study: a randomized trial. Jama 2007;297:969–77. 17341711 10.1001/jama.297.9.969

[24] Shai I Schwarzfuchs D Henkin Y Weight loss with a low-carbohydrate, Mediterranean, or low-fat diet. N Engl J Med 2008;359:229–41. 18635428 10.1056/NEJMoa0708681

[25] Boyle MA Samaha AL Rodewald AM Evaluation of the reliability and validity of GraphClick as a data extraction program. Comput Hum Behav 2013;29:1023–7.

[26] Teixeira PJ Going SB Sardinha LB A review of psychosocial pre-treatment predictors of weight control. Obes Rev 2005;6:43–65. 15655038 10.1111/j.1467-789X.2005.00166.x

[27] Dias S Ades AE. Absolute or relative effects? Arm-based synthesis of trial data. Res Synth Methods 2016; 7:23–8. 26461457 10.1002/jrsm.1184 PMC5102631

[28] Salanti G Higgins JP Ades A Evaluation of networks of randomized trials. Stat Methods Med Res 2008;17: 279–301. 17925316 10.1177/0962280207080643

[29] Gleser LJ Olkin I. Stochastically dependent effect sizes In: Cooper H Hedges LV Valentine JCs (eds)*.* The Handbook of Research Synthesis and Meta-Analysis. New York: Russell Sage Foundation, 2009, 357–76.

[30] R Development Core Team. R: A Language and Environment for Statistical Computing. Vienna, Austria: R Foundation for Statistical Computing, 2015 Version 3.2.1. http://www.r-project.org/ (13 July 2016, date last accessed).

[31] Viechtbauer W. Conducting meta-analyses in R with the metafor package. J Stat Softw 2010;36:1–48.

[32] Senior AM Gosby A Lu J Simpson SJ Data from: Meta-Analysis of Variance: an Illustration Comparing the Effects of Two Dietary Interventions on Variability in Weight. In Evolution Medicine and Public Health. Dryad Data Repository. 2016. http://dx.doi.org/10.5061/dryad.337dr. 10.1093/emph/eow020 PMC4981479 27491895

[33] Brown H Prescott R. Multi-centre trials and meta-analyses In: Brown H Prescott Rs (eds)*.* Applied Mixed Models in Medicine. Chichester, UK: John Wiley & Sons Ltd, 2015, 197–230.

[34] Hedges LV Gurevitch J Curtis PS. The meta-analysis of response ratios in experimental ecology. Ecology 1999;80: 1150–6.

[35] Friedrich JO Adhikari NK Beyene J. The ratio of means method as an alternative to mean differences for analyzing continuous outcome variables in meta-analysis: a simulation study. BMC Med Res Methodol 2008;8:32. 18492289 10.1186/1471-2288-8-32 PMC2430201

[36] Blyth CR. On Simpson’s Paradox and the Sure-Thing Principle. J Am Stat Assoc 1972;67:364–6.

[37] Simpson SJ Raubenheimer D. Obesity: the protein leverage hypothesis. Obes Rev 2005;6:133–42. 15836464 10.1111/j.1467-789X.2005.00178.x

[38] Gosby AK Conigrave AD Lau NS Testing Protein Leverage in Lean Humans: a Randomised Controlled Experimental Study. PLoS One 2011;6:e25929. 22022472 10.1371/journal.pone.0025929 PMC3192127

[39] Martinez-Cordero C Kuzawa CW Sloboda DM Testing the Protein Leverage Hypothesis in a free-living human population. Appetite 2012;59:312–5. 22634200 10.1016/j.appet.2012.05.013

[40] Larsen TM Dalskov S-M van Baak M Diets with high or low protein content and glycemic index for weight-loss maintenance. N Engl J Med 2010;363:2102–13. 21105792 10.1056/NEJMoa1007137 PMC3359496

[41] Campbell CP Raubenheimer D Badaloo AV Developmental contributions to macronutrient selection: A randomized controlled trial in adult survivors of malnutrition. Evol Med Public Health 2016;2016:158–69. 26817484 10.1093/emph/eov030 PMC4871598

[42] Solon-Biet Samantha M McMahon Aisling C Ballard JWilliam O The ratio of macronutrients, not caloric intake, dictates cardiometabolic health, aging, and longevity in ad libitum-fed mice. Cell Metab 2014;19:418–30. 24606899 10.1016/j.cmet.2014.02.009 PMC5087279

[43] Le Couteur D Solon-Biet S Cogger V The impact of low-protein high-carbohydrate diets on aging and lifespan. Cell Mol Life Sci 2016;76:1237–52. 26718486 10.1007/s00018-015-2120-y PMC11108352

[44] Solon-Biet SM Mitchell SJ de Cabo R Macronutrients and caloric intake in health and longevity. J Endocrinol 2015; 226:R17–28. 26021555 10.1530/JOE-15-0173 PMC4490104

[45] Sacks FM Bray GA Carey VJ Comparison of Weight-Loss Diets with Different Compositions of Fat, Protein, and Carbohydrates. N Engl J Med 2009;360: 859–73. 19246357 10.1056/NEJMoa0804748 PMC2763382

[46] Dansinger ML Gleason JA Griffith JL Comparison of the Atkins, Ornish, Weight Watchers, and Zone diets for weight loss and heart disease risk reduction: a randomized trial. Jama 2005;293:43–53. 15632335 10.1001/jama.293.1.43

[47] Foster GD Wyatt HR Hill JO A randomized trial of a low-carbohydrate diet for obesity. N Engl J Med 2003;348:2082–90. 12761365 10.1056/NEJMoa022207

[48] Foster GD Wyatt HR Hill JO Weight and metabolic outcomes after 2 years on a low-carbohydrate versus low-fat diet: a randomized trial. Ann Intern Med 2010;153:147–57. 20679559 10.1059/0003-4819-153-3-201008030-00005 PMC2949959

[49] Iqbal N Vetter ML Moore RH Effects of a low-intensity intervention that prescribed a low-carbohydrate vs. a low-fat diet in obese, diabetic participants. Obesity 2010;18:1733–8. 20019677 10.1038/oby.2009.460

[50] Samaha FF Iqbal N Seshadri P A low-carbohydrate as compared with a low-fat diet in severe obesity. N Engl J Med 2003;348:2074–81 12761364 10.1056/NEJMoa022637

