# Supplementary material for: Meta-analysis of variance: an illustration comparing the effects of two dietary interventions on variability in weight
Source: Evol Med Public Health. 2016 Aug 3;2016(1):244–55. doi: 10.1093/emph/eow020 (PMC4981479; doi:10.1093/emph/eow020)
Supplement: Supplementary Data [file supp_2016_1_244__index.html]

Meta-analysis of variance: an illustration comparing the effects of two dietary interventions on variability in weight — Supplementary Data 

# Meta-analysis of variance: an illustration comparing the effects of two dietary interventions on variability in weight

## Supplementary Data

files

- Supplementary Data - zip file
