# Supplementary material for: Meta-analysis of variance: an illustration comparing the effects of two dietary interventions on variability in weight
Source: Evol Med Public Health. 2016 Aug 3;2016(1):244–55. doi: 10.1093/emph/eow020 (PMC4981479; doi:10.1093/emph/eow020)
Supplement: Supplementary Data [file supp_eow020_suppl_data.zip › Supplementary_File_2_Supplementary_Results.docx]

# Supplementary Results for Meta-analysis of variance: an illustration comparing the effects of two dietary interventions on variability in weight

Alistair M Senior^1,2,#^, Alison K Gosby^1,3^, Jing Lu^1^, Stephen J. Simpson^1,3^ and David Raubenheimer^1,3,4^

1. Charles Perkins Centre, The University of Sydney, Sydney, New South Wales, 2006, Australia
2. School of Mathematics and Statistics, The University of Sydney, Sydney, New South Wales, 2006, Australia
3. School of Life and Environmental Sciences, The University of Sydney, Sydney, New South Wales, 2006, Australia
4. Faculty of Veterinary Sciences, The University of Sydney, Sydney, New South Wales, 2006, Australia

# Corresponding Author

## Table S1

Results from multi-level meta-analysis of lnRR, lnVR and lnCVR. From each meta-analysis is shown the Estimate, the upper and lower 95% confidence limit (LCL and UCL), the variance components (*σ*2) for the random-effects fitted to the model, the *Q* statistic for total heterogeneity, and the associated p-value. Estimates with a lower to upper confidence limit (LCL, UCL) not spanning zero are considered statistically significant.

| Response | Estimate | LCL | UCL | *σ*^2^_Article_ | *σ*^2^_Resdicual_ | *Q*_Total_ | *p*_Q_ |
| --- | --- | --- | --- | --- | --- | --- | --- |
| lnRR | 0.02 | -0.01 | 0.04 | 0.00 | 0.00 | 2.97 | 0.94 |
| lnVR | -0.08 | -0.19 | 0.02 | 0.00 | 0.01 | 9.84 | 0.28 |
| lnCVR | -0.10 | -0.20 | 0.90 $\times$ 10^-3^ | 0.00 | 0.01 | 15.61 | < 0.05 |

## Table S2.

Results from multi-level meta-regression fitting ln$\bar{x}$ and lnSD as responses, and diet type (calorie restricted or carbohydrate restricted) as moderator variables, and the variance components (*σ*^2^) for the random-effects fitted to the model (where the *σ*^2^_Article_ is non-zero the estimated correlation between-group within-article correlation is given in brackets). In the case of lnSD, mean mass [Z transformed; 1] was also fitted as a moderator variable. Estimates with a lower to upper confidence limit (LCL, UCL) not spanning zero are considered statistically significant.

| Response | Fixed Coefficients | Estimate | LCL | UCL | *σ*^2^_Article_ | *σ*^2^_Resdiual_ |
| --- | --- | --- | --- | --- | --- | --- |
| ln$\bar{x}$ | (*β*_0_) Intercept _LC_ | 4.56 | 4.45 | 4.68 | 0.02 (1.00) | 0.00 |
|  | (*β*_1_) Difference _CR – LC_ | 0.02 | -0.01 | 0.04 |  |  |
| lnSD | (*β*_0_) Intercept _LC_ | 2.88 | 2.80 | 2.95 | 0.00 | 0.70 $\times$ 10^-3^ |
|  | (*β*_1_) Difference _CR – LC_ | -0.11 | -0.21 | -0.02 |  |  |
|  | (*β*_2_) *Z* Mean Mass (Kg) | 0.20 | 0.14 | 0.26 |  |  |

## References

1. Schielzeth H; Simple means to improve the interpretability of regression coefficients. *Methods Ecol Evol* 2010;**1**(2):103-113. doi: 10.1111/j.2041-210X.2010.00012.x.
