# Supplementary material for: Meta-analysis of variance: an illustration comparing the effects of two dietary interventions on variability in weight
Source: Evol Med Public Health. 2016 Aug 3;2016(1):244–55. doi: 10.1093/emph/eow020 (PMC4981479; doi:10.1093/emph/eow020)
Supplement: Supplementary Data [file supp_eow020_suppl_data.zip › Supplementary_File_1_Supplementary_Methods.docx]

# Supplementary Methods for Meta-analysis of variance: an illustration comparing the effects of two dietary interventions on variability in weight

Alistair M Senior^1,2, #^, Alison K Gosby^1,3^, Jing Lu^1^, Stephen J. Simpson^1,3^ and David Raubenheimer^1,3,4^

1. Charles Perkins Centre, The University of Sydney, Sydney, New South Wales, 2006, Australia
2. School of Mathematics and Statistics, The University of Sydney, Sydney, New South Wales, 2006, Australia
3. School of Life and Environmental Sciences, The University of Sydney, Sydney, New South Wales, 2006, Australia
4. Faculty of Veterinary Sciences, The University of Sydney, Sydney, New South Wales, 2006, Australia

# Corresponding Author

A fundamental assumption of meta-analytic models is that effect sizes are independent from one another. This assumption can be violated when effect sizes are calculated as contrasts between several treatment groups and a single control group from the same study; sometimes terms stochastic dependency [1]. This was the case for two studies in our dataset, which contained multiple groups on a calorie restricted (CR) diet, and one group on a low carbohydrate *ad libitum* (LC) diet.

Independence of this nature can be modelled in *metafor* using the rma.mv by including a matrix that contains the estimated covariance between effect sizes that share data [2]. The covariance between instances of lnRR and lnCVR that share control data was estimated by the methods outlined in Lajeunesse [3] and Senior, Nakagawa [4]. For lnVR, the covariance between instances that are based on the same LC groups was estimated as equation S1:

 eq. S1,

where *n* is the sample size of the LC group common to both effect sizes. The covariance between instance of lnCVR that are based on lnCVR was estimated as equation S2:

 eq. S2,

where *n, s* $\bar{x}$ is the sample size, standard deviation and mean of the LC group common to both effect sizes, respectively, and *ρ* is the correlation between ln$\bar{x}$ and lnSD.

## References

1. Gleser LJ, Olkin I; Stochastically dependent effect sizes. In: Cooper H, Hedges LV, Valentine JCs (eds)*. The Handbook of Research Synthesis and Meta-Analysis*. New York: Russell Sage Foundation, 2009, 357-376.

2. Viechtbauer W; Conducting meta-analyses in R with the metafor package. *J Stat Softw* 2010;**36**:1-48. doi: 10.18637/jss.v036.i03.

3. Lajeunesse MJ; On the meta-analysis of response ratios for studies with correlated and multi-group designs. *Ecology* 2011;**92**(11):2049-2055. doi: 10.1890/11-0423.1.

4. Senior AM, Nakagawa S, Lihoreau M, et al.; An overlooked consequence of dietary mixing: a varied diet reduces inter-individual variance in fitness. *Am Nat* 2015;**186**(5):649-659. doi: 10.1086/683182.
